# *Announcement*: World IBD Day — May 19, 2017

**DOI:** 10.15585/mmwr.mm6619a9

**Published:** 2017-05-19

World IBD Day is recognized on May 19 to raise awareness of inflammatory bowel disease (IBD) and the two conditions that comprise it: Crohn’s disease and ulcerative colitis, both of which cause chronic inflammation of the gastrointestinal tract. World IBD Day is sponsored by the European Federation of Crohn’s and Ulcerative Colitis Associations, which includes the Crohn’s & Colitis Foundation.

In 2015, approximately 3 million adults in the United States self-reported having a diagnosis of IBD, representing a large increase from <2 million in 1999 (*1*,*2*). Symptoms can include frequent diarrhea, abdominal pain, and bloody stools, as well as fever, weight loss, fatigue, and night sweats. The cause of IBD is not known, but genetic susceptibilities, problems with the immune system, and environmental exposures, such as smoking and certain microorganisms, all might play a role. Although to date, IBD cannot be prevented, there are ways to manage the symptoms and prevent complications. Additional information is available at https://www.cdc.gov/ibd/. Additional information on World IBD Day is available from the Crohn’s & Colitis Foundation (http://www.crohnscolitisfoundation.org/WorldIBDDay/).
